# Profiling of *O*-acetylated Gangliosides Expressed in Neuroectoderm Derived Cells

**DOI:** 10.3390/ijms21010370

**Published:** 2020-01-06

**Authors:** Sumeyye Cavdarli, Nao Yamakawa, Charlotte Clarisse, Kazuhiro Aoki, Guillaume Brysbaert, Jean-Marc Le Doussal, Philippe Delannoy, Yann Guérardel, Sophie Groux-Degroote

**Affiliations:** 1 Univ. Lille, CNRS, UMR 8576–UGSF-Unité de Glycobiologie Structurale et Fonctionnelle, F-59000 Lille, France; sumeyye.cavdarli@univ-lille.fr (S.C.); nao.yamakawa@univ-lille.fr (N.Y.); charlotte.clarisse@univ-lille.fr (C.C.); guillaume.brysbaert@univ-lille.fr (G.B.); philippe.delannoy@univ-lille.fr (P.D.); yann.guerardel@univ-lille.fr (Y.G.); 2 OGD2 Pharma, Institut de Recherche en Santé de l’Université de Nantes, 44007 Nantes, France; ledoussal@ogd2pharma.com; 3 Complex Carbohydrate Research Center, University of Georgia, Athens, GA 30602, USA; kaoki@ccrc.uga.edu

**Keywords:** gangliosides, *O*-acetylation, mass spectrometry, neuroectoderm-derived cancer, sialic acid

## Abstract

The expression and biological functions of oncofetal markers GD2 and GD3 were extensively studied in neuroectoderm-derived cancers in order to characterize their potential as therapeutic targets. Using immunological approaches, we previously identified GD3, GD2, and *O*AcGD2 expression in breast cancer (BC) cell lines. However, antibodies specific for *O*-acetylated gangliosides are not exempt of limitations, as they only provide information on the expression of a limited set of *O*-acetylated ganglioside species. Consequently, the aim of the present study was to use structural approaches in order to apprehend ganglioside diversity in melanoma, neuroblastoma, and breast cancer cells, focusing on *O*-acetylated species that are usually lost under alkaline conditions and require specific analytical procedures. We used purification and extraction methods that preserve the *O*-acetyl modification for the analysis of native gangliosides by MALDI-TOF. We identified the expression of GM1, GM2, GM3, GD2, GD3, GT2, and GT3 in SK-Mel28 (melanoma), LAN-1 (neuroblastoma), Hs 578T, SUM 159PT, MDA-MB-231, MCF-7 (BC), and BC cell lines over-expressing GD3 synthase. Among *O*-acetylated gangliosides, we characterized the expression of *O*AcGM1, *O*AcGD3, *O*AcGD2, *O*AcGT2, and *O*AcGT3. Furthermore, the experimental procedure allowed us to clearly identify the position of the sialic acid residue that carries the *O*-acetyl group on b- and c-series gangliosides by MS/MS fragmentation. These results show that ganglioside *O*-acetylation occurs on both inner and terminal sialic acid residue in a cell type-dependent manner, suggesting different *O*-acetylation pathways for gangliosides. They also highlight the limitation of immuno-detection for the complete identification of *O*-acetylated ganglioside profiles in cancer cells.

## 1. Introduction

**Figure 1:**
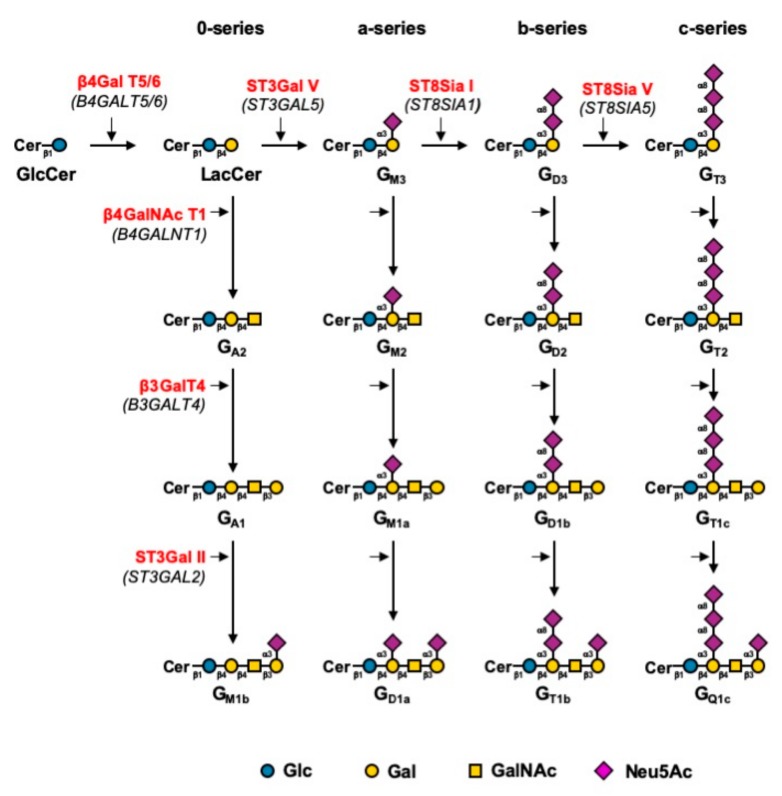
Biosynthesis pathway for gangliosides [2]. Gangliosides are synthesized by stepwise addition of monosaccharides to GlcCer. Extension of GlcCer occurs through the action of the UDP-Gal: GlcCer β1,4-galactosyltransferase to make lactosylceramide (LacCer). The action of the GM3 synthase, GD3 synthase, and GT3 synthase leads to the biosynthesis of the precursors of a-, b-, and c-series gangliosides, respectively. The 0-series gangliosides are directly synthesized from LacCer. Elongation is performed by the sequential action of β4GalNAc T1, β3Gal T4, and the sialyltransferases ST3Gal II and ST8Sia V. Genes encoding the different glycosyltransferases involved are indicated in italics between brackets.

Gangliosides are acidic glycosphingolipid carrying one or several sialic acid residues located in lipid raft in which they play an important role in the regulation of cell signaling [1]. Their biosynthesis process has been well established as depicted in Figure 1 [2]. Indeed, lipid raft domains are microdomains from which major cellular pathways are engaged, not only in normal physiological conditions, but also under pathological conditions, such as neurodegenerative diseases or cancer. In neuroectoderm-derived cancers, gangliosides over-expression participates to tumor aggressiveness. For example, in MDA-MB-231 breast cancer cell line, GD2 over-expression increases tumorigenesis by activating c-Met receptor [3]. These properties led to the characterization of gangliosides as tumor associated carbohydrate antigens (TACA). GD3 and GD2 have been described as TACA and also oncofetal markers in melanoma, small cell lung carcinoma (SCLC), and neuroblastoma [4,5], and are potent targets for immunotherapy, especially GD2. Therapeutic antibodies developed against GD2 have been approved by the Food Drug Administration (FDA) as dinutuximab and European Medicine Agency (EMA) as qarziba for the treatment of high-risk pediatric neuroblastoma [6]. The standard care for neuroblastoma is the use of intense chemotherapy to achieve clinical remission. However, the complete remission of patients is hard to achieve and relapse occurs in 50% of cases. The use of anti-GD2 therapeutic antibodies for relapsing neuroblastoma has shown promising results in patients, but also severe side toxicities such as allodynia [6]. Indeed, GD2 expression is not exclusive to cancer cells, and peripheral nerve fibers are expressing GD2, which explains the downside effects. Interestingly, the use of an antibody targeting specifically the *O*-acetylated form of GD2 (*O*AcGD2) suggested that *O*AcGD2 expression is exclusive to cancer cells and tissues [7]. Terme et al. have shown that 8B6 antibody targeting *O*AcGD2 exhibits the same efficiency as anti-GD2 antibody without inducing allodynia [8]. Besides, *O*-acetylated GD2 has been shown to promote neuroblastoma growth in vitro, and in vivo [8,9]. Similarly, *O*-acetylated GD3 (*O*AcGD3) protects glioblastoma cells from GD3 induced mitochondrial apoptosis [10,11]. These properties exhibited by the *O*-acetylated forms of GD3 and GD2 pose them as valuable alternative therapeutic targets compared to their non-*O*-acetylated forms. However, there is very limited knowledge about ganglioside *O*-acetylation mechanisms. Previous studies using immunodetection analysis established that breast cancer cell lines displayed major expression of GD3, GD2, and *O*AcGD2, but not *O*AcGD3 [12]. Baumann and coworkers have previously reported that CASD1 was the only human sialyl*-O*-acetyltransferase (SOAT), and suggested it would act on the sialic acid donor CMP-Neu5Ac rather than on glycolipid itself [13]. The *O*-acetylation of gangliosides catalyzed by SOAT may occur on the hydroxyl residues of C4, C7, C8, or C9. In contrast, our work suggests that the *O*AcGD2 is generated directly from GD2 in breast cancer cells [12]. In order to get insights into the biosynthesis mechanisms underlying ganglioside *O*-acetylation process, we decided to establish the pattern of *O*-acetylated gangliosides of neuroectoderm-derived cell lines that express tumor-associated GD2 and GD3, with a special interest for *O*AcGD3 and *O*AcGD2. 

Indeed, despite the biological relevance of *O*-acetylated GSL in cancer, very little is known about their distributions in cells and tissues and about their fine chemical structure, i.e., the position of the *O*-acetyl group(s), and which sialic acid residue is *O*-acetylated in di- or tri-sialylated ganglioside species. This is partly related to the fact that detection and analysis of *O*-acetylated gangliosides remain challenging due to the lability of the *O*-acetyl group using classical procedures and to the lack of specific antibodies that show minimal cross-reactivity with non-acetylated glycan moieties [14,15,16]. In this context, we established the expression patterns of gangliosides in neuroectoderm-derived cancers cells using mass spectrometry. To this end, we combined a purification and extraction procedure for native gangliosides analysis by MALDI-TOF, avoiding the loss of the *O*-acetyl modifications. GM1, GM2, GM3, GD2, GT2, and GT3 gangliosides have been identified in SK-Mel-28 melanoma cells, in LAN-1 neuroblastoma cells, in Hs 578T, SUM159PT, MDA-MB-231, MCF-7 BC cells, and in BC cell lines over-expressing GD3 synthase. Among *O*-acetylated gangliosides, we characterized the expression of *O*AcGM1, *O*AcGD3, *O*AcGD2, *O*AcGT3, *O*AcGT2. This method allowed us to identify the sialic acid residues carrying the *O*-acetyl groups on b- and c-series gangliosides by MS/MS fragmentation, giving us insights into the cellular *O-*acetylation mechanisms.

## 2. Results

### 2.1. O-acetylated Sialic Acid Species Are Highly Expressed on Glycolipids in Cancer Cells

**Figure 2:**
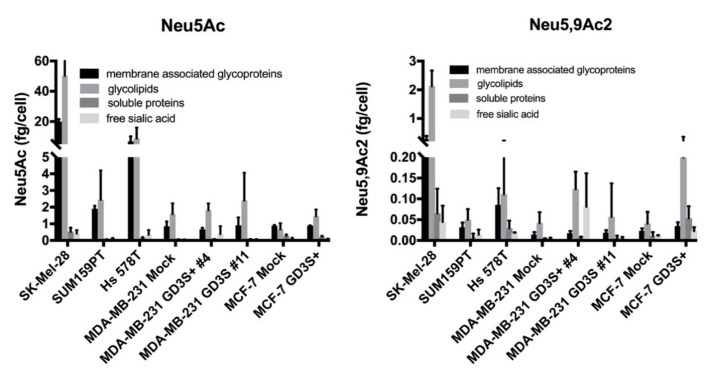
Neu5Ac and Neu5,9Ac_2_ DMB-Sia derivatives quantification by LC-ESI/MS in different cell fractions. Cell pellets were fractionated into membrane associated glycoproteins, glycolipids, soluble proteins, and free sialic acid fractions and hydrolyzed by propionic acid. The extracted sialic acids were derivatized using DMB and injected into LC-ESI/MS for identification and quantification. The amounts of Neu5Ac and Neu5,9Ac_2_ were quantified by integrating the corresponding areas obtained using extracted ion chromatogram on DMB-Sia derivatives. Each bar represents the mean of three independent experiments.

As previously described, we performed the quantification of sialic acid species in cancer cells using LC-ESI/MS analysis of DMB-Sia derivatives [12]. Our results suggest that the main *O*-acetylated sialic acid species expressed by these cells is Neu5,9Ac_2_. Here, we quantified the amounts of total sialic acid (Neu5Ac) and acetylated sialic acid (Neu5,9Ac_2_) in different cell fractions. Dried pellets were fractionated into membrane associated glycoproteins, soluble proteins, glycolipids, and free sialic acid before sialic acid extraction and DMB derivatization. As depicted in Figure 2, the amounts of Neu5Ac remain higher in all fractions compared to Neu5,9Ac_2_ levels, reaching 60 fg/cell and 3 fg/cell, respectively. 

While the amounts of Neu5Ac and Neu5,9Ac_2_ were markedly cell-type dependent, the highest amount of sialic acid was quantified in the glycolipid fraction, except for the MDA-MB-231 GD3S+ clone #4, which exhibited high expression of Neu5,9Ac_2_ in the free sialic acid fraction. Besides the glycolipid fraction, the highest expression of Neu5,9Ac_2_ was detected in the membrane associated glycoprotein fraction for SK-MEL-28, MDA-MB-231 GD3S+ clones #4 and #11, MCF-7 GD3S+, progressively decreasing in Hs 578T, SUM159PT, MDA-MB-231, and MCF-7 (Figure 2). These results complete our previous study, confirming that GD3 synthase over-expression increases sialic acid *O*-acetylation. In order to supplement this approach and to identify the *O*-acetylated ganglioside species, native gangliosides extracted from all cell lines were analyzed by MALDI-QIT-TOF.

### 2.2. Profiling of O-acetylated Gangliosides in Breast Cancer, Melanoma, and Neuroblastoma Cell Lines

**Figure 3:**
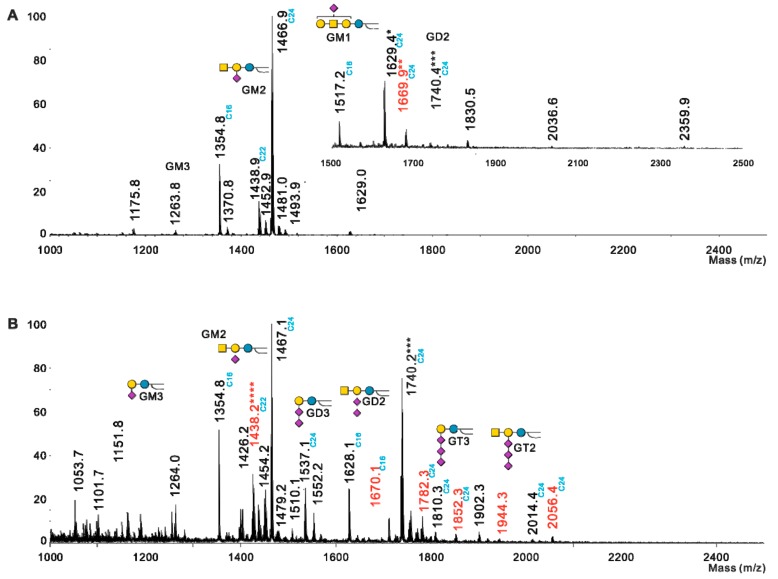
Gangliosides profiling by representative MS spectra in Hs 578T (**A**) and MDA-MB-231 GD3S+#4 (**B**). Ceramide consists mainly of d18:1 long chain base and C16, C18, C24 either/both saturated or/and saturated fatty acids. Red signals identify acetylated gangliosides species and black signals non-acetylated forms of gangliosides species. The nature of long fatty acid chain is indicated in blue on the spectra. * and *** indicates that GD2 Cer^16^ and *O*AcGT3Cer^16^ are respectively present in traces amount; ** indicates that signal corresponds to a mixture of *O*AcGM1Cer^24^ with *O*AcGD2Cer^18^; **** indicates that signal corresponds to a mixture of GM2 with *O*AcGD3. Gangliosides are depicted according to the following 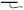 Ceramide; 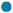 Galactose; 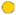 Glucose; 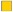 *N*-acetyl-galactosamine; 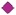 *N*-acetyl-neuraminic acid.

The detection of *O*-acetylated gangliosides by mass spectrometry is a challenging issue due to the loss of *O*-acetyl groups following chemical treatments usually performed to improve purification (mild saponification of non-ceramide lipids) or detection sensibility in mass spectrometry analysis (permethylation). Here, we used extraction and purification methods designed to preserve *O*-acetylation of GSL. Potentially *O*-acetylated GSL were extracted from dried pellets on eight different neuroectoderm-derived cell lines and analyzed in native forms by MALDI-QIT-TOF. Ganglioside expression patterns were established based on the calculated compositions of individual signals and further confirmed by MS/MS analyses. It is noteworthy that most di- and tri-sialylated GSL were observed mostly at *m*/*z* [M−H2O−H]^−^ and [M−2H_2_O−H]^−^ adducts, respectively (Table 1). MS/MS fragmentation analyses showed that dehydration of GSL resulted from the lactonization of oligosialic acids [17]. Examples of spectra obtained in Hs 578T and MDA-MB-231 GD3S+ clone #4 are presented in Figure 3. Mass spectra obtained for Hs 578T BC cell line led to the identification of GM3, GM2, GM1, *O*AcGM1, *O*AcGD2, and of trace amounts of GD2, *O*AcGD3, and *O*AcGT3 (Figure 3A). In MDA-MB-231 GD3S+ clone #4, GM3, GM2, GM1, GD3, GD2, GD1b, GT3, GT2, *O*AcGD3, *O*AcGD2, and *O*AcGT3 were detected (Figure 3B). These two cell lines exhibit similar expression patterns of gangliosides, but a cell-type dependent pattern of *O*-acetylated gangliosides species (Figure 3). These two cell lines exhibit similar expression patterns of gangliosides, but a cell-type dependent pattern of *O*-acetylated gangliosides species (Figure 3).

**Figure 4:**
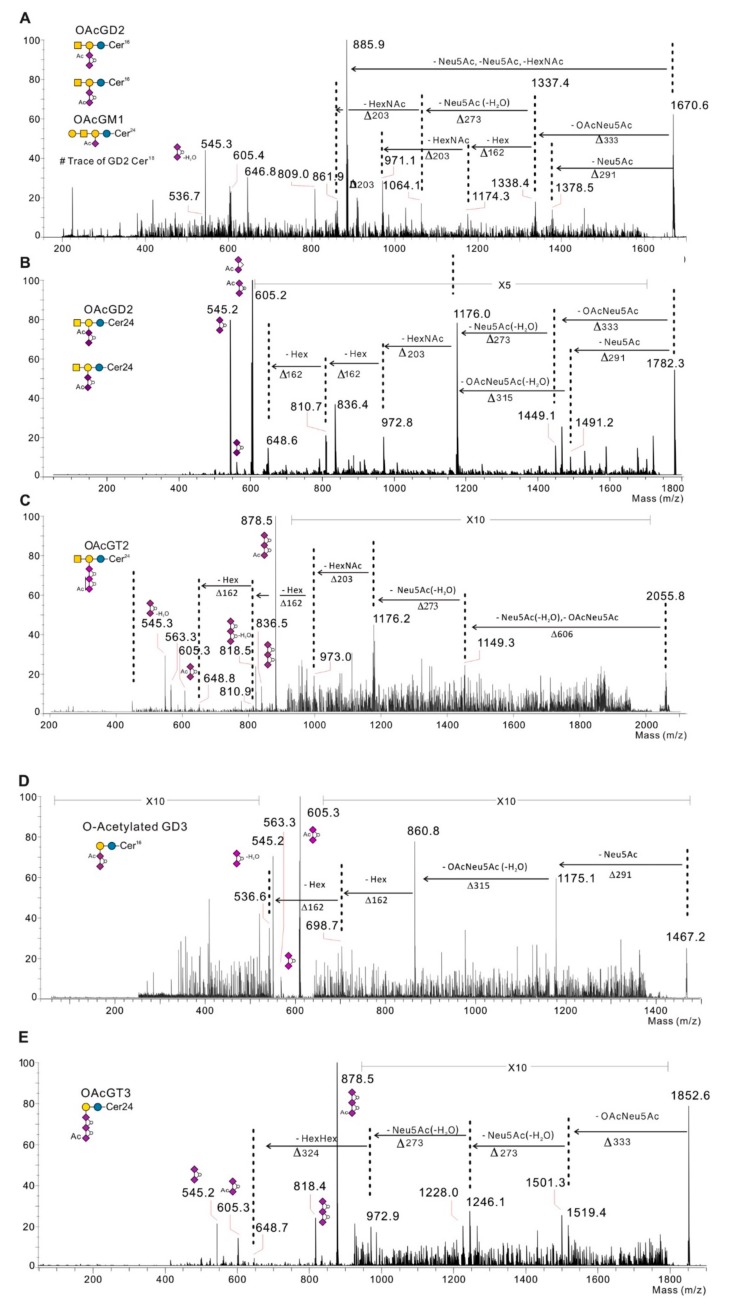
Representative MS/MS fragmentation of the molecular ion corresponding to *O*AcGM1, *O*AcGD3, *O*AcGD2, *O*AcGT3, and *O*AcGT2. (**A**) MS/MS fragmentation of the molecular ion *m*/*z* 1670 in Hs 578T corresponding to *O*AcGM1 and *O*AcGD2 ganglioside. MS/MS fragmentation of the molecular ion *m*/*z* 1782 (**B**) *m*/*z* 2055 (**C**) respectively corresponding to *O*AcGD2 and *O*AcGT2 gangliosides in MDA-MB-231 GD3S + clone #4. (**D**) MS/MS fragmentation of the molecular ion *m*/*z* 1467 corresponding to *O*AcGD3 identified in SK-Mel-28. (**E**) MS/MS fragmentation of the molecular ion *m*/*z* 1852 corresponding to *O*AcGT3 identified in MDA-MB-231 GD3S+ clone #11. Gangliosides are indicated according to the following 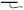 Ceramide; 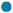 Galactose; 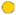 Glucose; 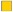 *N*-acetyl-galactosamine; 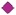 *N*-acetyl-neuraminic acid, ° dehydration Ac: *O*-acetyl group.

As depicted in Figure 4, MS/MS fragmentation has been performed for *O*-acetylated gangliosides so as to confirm signal identification, especially when one signal might correspond to two isobaric structures. Moreover, in the case of the identification of two species separately for one molecular ion by MS/MS fragmentation, the fragmentation allows the comparison of the relative amount of the two species characterized. For example, the fragmentation of the signal at *m*/*z* 1670 in Hs 578T confirmed the identification of the following two species: *O*AcGD2 (d18:1 Cer16:0) and *O*AcGM1 (d18:1 Cer24:0) (Figure 4A).

**Figure 5:**
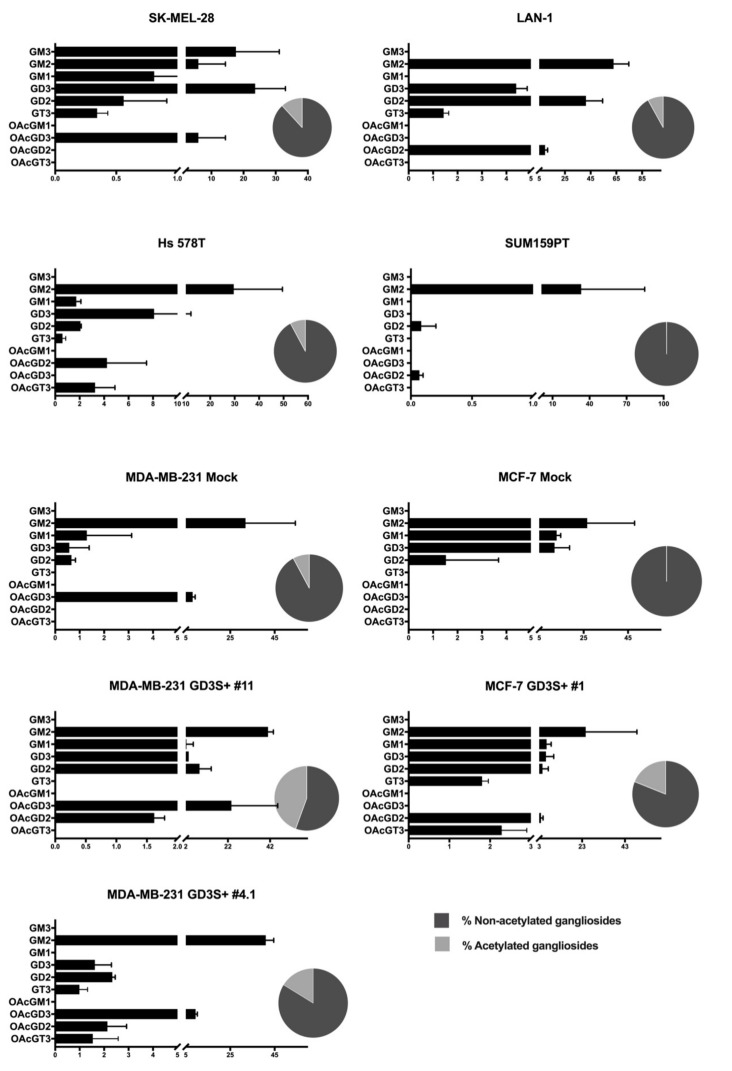
Relative quantification of the global content of main ganglioside species by MALDI-QIT-TOF. Relative quantification of ganglioside content of the apex intensities (mV) of the peak assigned on MALDI-QIT-TOF spectra (*n* = 3). Total ganglioside content was normalized to 100 for each cell line. The relative amount of each species is calculated as the percentage of the total ganglioside content. Pie charts represent the percentage of total acetylated gangliosides (light grey) vs. non-acetylated (dark grey).

As depicted in Table 1, our results indicate that the pattern of ganglioside *O*-acetylation is not representative of neuroectoderm derived cancer cells, but is cell type-dependent, while the representative pattern of expressed gangliosides is similar between the different cell lines. Basically, GM1, GM2, GM3, and GD2 are expressed in all cell lines tested. GD3 is expressed in SK-Mel-28, LAN1, MDA-MB-231 GD3S+ clone #4 and #11. GT3 and GT2 are expressed in MDA-MB-231 GD3S+ clone #4 and #11. Regarding *O*-acetylated gangliosides species pattern in cancer cell lines, *O*AcGD2 is expressed in all cell lines except SK-Mel-28, MDA-MB-231, and MCF-7. *O*AcGD3 is expressed in SK-Mel-28, LAN-1, Hs 578T, MDA-MB-231 GD3S+ clone #11, and MCF-7 GD3S+ clone #1. *O*AcGT3 and *O*AcGT2 are in turn expressed in MDA-MB-231 GD3 synthase overexpressing clones (Figure 5). The method used here led us to estimate the structure of sphingoid base and fatty acid chain according to the *m*/*z* detected for all ganglioside species identified. Most gangliosides exhibit a ceramide moiety corresponding to the combination of a long chain base sphingosine d18:1 and a fatty acid chain composed mainly by C16 or C24. C14, C18, and C22 are detected as minor components in cells. MCF-7 cell line displays variations on ceramide moiety as mixture of different combinations of the sphingosine base d18:1 and a highly hydroxylated ceramide (Supplementary Figure S1). Besides, C16 and C18 fatty acids are saturated, whereas C24 is always present in its saturated and unsaturated form (data not shown).

### 2.3. O-acetylated Ganglioside Species Expression Increases in GD3 Synthase Overexpressing Clones

The relative amounts of the different ganglioside species were calculated by integrating the intensity of individual signals detected on MALDI-QIT-TOF mass spectra. The proportion of *O*-acetylated gangliosides was 1% in SUM159PT, 9% in MDA-MB-231 and MCF-7, 10% in LAN-1 and Hs 578T, and 12% in SK-Mel-28 (Figure 5). The highest amounts of *O*-acetylated gangliosides were observed in clones over-expressing GD3 synthase in a cell dependent manner: 18% in MDA-MB-231 GD3S+ clone #4, 22% in MCF-7 GD3S+, and 50% in MDA-MB-231 GD3S+ clone #11. In parallel, we observed that *O*AcGD3 was the most expressed *O*-acetylated ganglioside species compared to *O*AcGD2 and *O*AcGT3. *O*AcGD3 expression was quantified as 23.6% of total gangliosides in MDA-MB-231 GD3S+ clone #11, 9.5% in MDA-MB-231 GD3S+ clone #4, 9% in MDA-MB-231 mock, 5.9% in SK-MEL-28. *O*AcGD2 expression remains lower than *O*AcGD3 content in all cell lines.

Besides the diversity observed for the position of the *O*-acetyl group on sialic acid, the *O-*acetylated ganglioside species expressed is cell line-dependent. *O*AcGD3 is the only *O*-acetylated ganglioside expressed by SK-Mel-28, MDA-MB-231, while *O*AcGD2 is solely expressed in LAN-1 and SUM159PT cells. In the other cancer cell lines, different combinations of *O*-acetylated gangliosides species are detected: *O*AcGD2 and *O*AcGT3 are detected in Hs 578T and MCF-7 GD3S+. MDA-MB-231 clone #11 express both *O*AcGD3 and *O*AcGD2, whereas MDA-MB-231 clone #4 shows the additional expression of *O*AcGD3, *O*AcGD2, and *O*AcGT3. Interestingly, GD3 synthase over-expression in MDA-MB-231 does not result in the expression of the same *O*-acetylated species expressed by the two clones. Moreover, *O*AcGD2 was not detected in MDA-MB-231 and MCF-7, but only in their clones over-expressing GD3 synthase, with a respective 2 and 3 fold increase, confirming our previous results [12]. *O*AcGT3 was the *O*-acetylated ganglioside species present in lowest amounts and was mostly present in Hs 578T (3.2%) and MDA-MB-231 clone #4 (1.5%) (Figure 5). *O*AcGT2 expression was confirmed using MS/MS fragmentation in MDA-MB-231 GD3S+ clone #4. However, signals assigned for *O*AcGT2 were too weak and could not be included in the relative quantification process.

### 2.4. Mapping of GTs Gene Expression and Gangliosides Content on a Ganglioside Metabolism Patway

**Figure 6:**
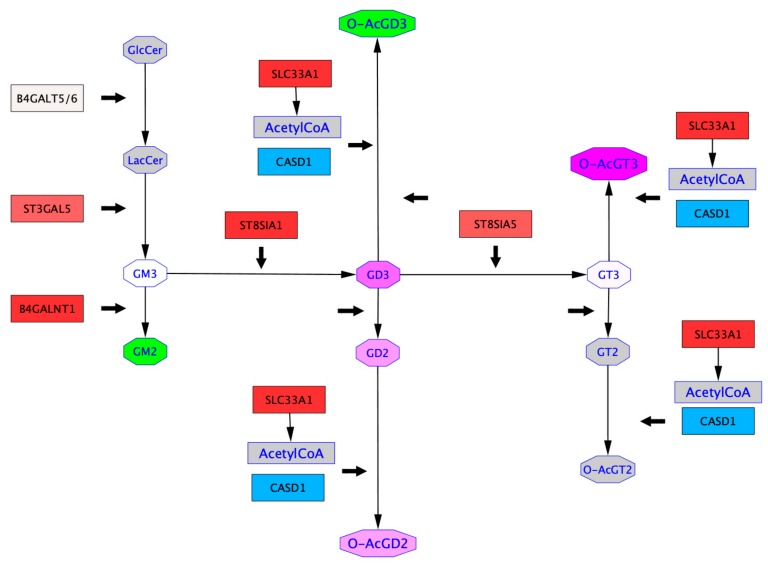
Differential ganglioside metabolism pathways between Hs 578T and MDA-MB-231 breast cancer cells. Glycosyltransferase gene expression profile obtained by qPCR were mapped onto a subpart of the “Ganglio-sphingolipid metabolism” pathway from WikiPathways [18,19] based on the differential expression between two cell lines. In the squared nodes, colors vary from blue (≤ −2) to white (= 0) and red (≥ 2) to indicate the repression to the over-expression of the glycosyltransferase gene in Hs 578T compared to MDA-MB-231 cells (thick black arrows). Quantitative data concerning the relative amounts of gangliosides obtained by MALDI-QIT-TOF mass spectrometry were added to the pathway based on the comparison between Hs 578T and MDA-MB-231 cells. In the octagonal nodes, colors vary from green (≤ −8) to white (= 0) and fuchsia (≥ 8) to indicate a restraint to a rise of the amount of a given ganglioside based on the differences observed between the two cell lines. Grey color indicates the absence of any available quantitative data about the expression.

**Figure 7:**
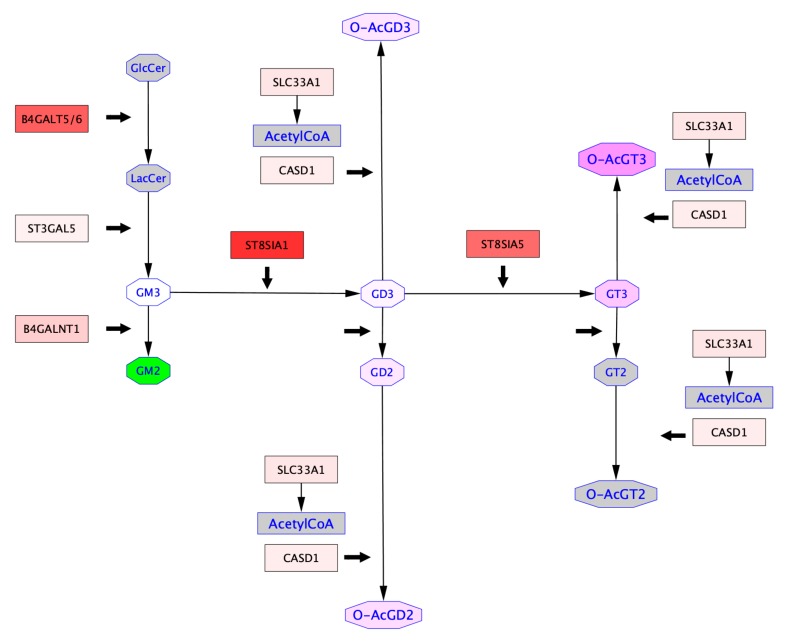
Differential ganglioside metabolism pathways between MDA-MB-231 GD3S+ clone #4 and MDA-MB-231 breast cancer cells. Glycosyltransferase gene expression profile obtained by qPCR were mapped onto a subpart of the “Ganglio-sphingolipid metabolism” pathway from WikiPathways [18,19] based on the differential expression between two cell lines. In the squared nodes, colors vary from blue (≤ −2) to white (= 0) and red (≥ 2) to indicate the repression to the over-expression of the glycosyltransferase gene in MDA-MB-231 GD3S+ clone #4 compared to MDA-MB-231 cells (thick black arrows). Quantitative data concerning the relative amounts of gangliosides obtained by MALDI-QIT-TOF mass spectrometry were added to the pathway based on the comparison between MDA-MB-231 GD3S+ clone #4 and MDA-MB-231 cells. In the octagonal nodes, colors vary from green (≤ −8) to white (= 0) and fuchsia (≥ 8) to indicate a restraint to a rise of the amount of a given ganglioside based on the differences observed between the two cell lines. Grey color indicates the absence of any available quantitative data about the expression.

In order to get an insight on ganglioside expression and biosynthesis mechanisms in cancer cells, we performed in silico analysis. Differential expression of genes encoding GTs and gangliosides were mapped onto a subpart of “Ganglio-sphingolipid metabolism” pathway retrieved from WikiPathway in Hs 578T vs. MDA-MB-231 cells (Figure 6) and in MDA-MB-231 vs. MDA-MB-231 GD3S+ cells (Figure 7). These representations ensure the combination of GTs gene expression and ganglioside content obtained respectively by qPCR and MALDI-QIT-TOF in one biosynthetic scheme. Thus, gene expression of *B4GALT6*, *ST3GAL5*, *ST8SIA1*, *B4GALNT1*, and *ST8SIA5* encoding GTs involved in gangliosides biosynthesis assessed by qPCR experiments in each cell line. Besides, *SCL33A1* and *CASD1* gene expression were analyzed by qPCR. These two genes were selected for their potential implication in ganglioside *O*-acetylation, respectively encoding the acetyl coenzyme 1 Golgi transporter and the only known human SOAT (Supplementary Figure S3). Our results highlight the upregulation of *ST3GAL5, B4GALNT1, ST8SIA1, ST8SIA5*, and *SCL33A1*, and the downregulation of *CASD1* in Hs 578T compared to MDA-MB-231 (Figure 6). All genes assessed by qPCR were up-regulated in MDA-MB-231 GD3S+ vs. MDA-MB-231 (Figure 7). These results highlight the repression of *CASD1* in Hs 578T compared to MDA-MB-231 cells, but the upregulation of *CASD1* in MDA-MB-231 GD3S+ compared to MDA-MB-231 cells.

Ganglioside proportions defined by MALDI-QIT-TOF analysis were also mapped onto these pathways to represent the differential ganglioside expression in Hs 578T vs. MDA-MB-231, and in MDA-MB-231 GD3S+ vs. MDA-MB-231. The differential ganglioside expression analysis brings out the upregulation of *O*AcGD2 and *O*AcGT3 and the downregulation of *O*AcGD3 in Hs578T compared to MDA-MB-231. In contrast, the same analysis highlights the upregulation of *O*AcGT3, *O*AcGD3, and *O*AcGD2 in MDA-MB-231 GD3S+ vs. MDA-MB-231 (Figure 7). These analyses reveal the upregulation of GD3, GD2, and GT3 in both pathways. The levels of gangliosides upregulation in Hs578T vs. MDA-MB-231 are higher than those identified in MDA-MB-231 GD3S+ vs. MDA-MB-231 (Figure 6 and Figure 7). The combinatorial approach that we used allows to perceive the relationships between gangliosides expressed and GTs gene expression between two cell lines. In both cases (Hs 578T vs. MDA-MB-231, and MDA-MB-231 GD3S+ vs. MDA-MB-231), *O*-acetylated gangliosides appear to be upregulated independently of *CASD1* expression variations between two cell lines, and in a substrate-dependent manner. *O*-acetylated ganglioside expression appears not only dependent on the expression level of enzymes involved in the biosynthesis of gangliosides, but also on substrate availability.

## 3. Discussion

For almost 50 years, profiling gangliosides expression in developmental and pathological conditions has been of high interest for their remarkable roles in oncogenesis and neurodegenerative diseases, in which gangliosides are considered as useful and promising diagnostic and prognostic tools, as well as targets for immunotherapy, especially in the field of neuroectoderm-related cancers (melanoma, neuroblastoma, small cell lung cancer) [20,21]. As an example, anti-GD2 clinical trials for neuroblastoma have confirmed the efficacy of anti-GD2 antibody immunotherapy for this rare but often lethal childhood cancer [22]. Different experimental approaches were developed to get more precise information about the structure of gangliosides from various biological samples. Basically, immunodetection methods and mass spectrometry are currently the major tools for ganglioside analysis. While immunodetection is based on in situ detection, mass spectrometry requires the extraction of gangliosides from biological samples before the analysis. Recently, the combination of these two detection methods has allowed a lot of progress regarding ganglioside profiling in sera [23,24], tissues [25], or cellular extracts [26]. Besides, *O*-acetylation of the sialic acid residues is one of the most common modifications of gangliosides and is sufficient to induce dramatic changes of physiopathological properties carried by the native non-*O*-acetylated gangliosides. For example, *O*-acetylation relocates GD3 from the mitochondria to the cell membrane, inducing the suppression of GD3 apoptotic effect in cancer cells [11,27,28]. Interestingly, targeting *O*AcGD2 rather than GD2 in cancer therapy seems to be a better option since *O*AcGD2 is exclusively expressed in cancer tissues [7].

The *O*-acetyl group is alkali labile, rendering it very difficult to handle for mass spectrometry analysis. Thus, immunodetection methods such as TLC, FACS, or immunocytochemistry followed by confocal microscopy were mainly used for the identification and quantification of *O*-acetylated gangliosides species [14]. Using the immunodetection methods mentioned above, we previously identified the expression of *O*AcGD2, but not *O*AcGD3 in BC cells [12]. However, the absence of *O*AcGD3 in BC cells was not confirmed in this work [12]. Indeed, native gangliosides extracted from the same cell lines were positive for *O*AcGD3 expression, indicating that the anti-*O*AcGD3 7H2 mouse IgG3 (Santa-Cruz biotechnology, Dallas, TX, USA) used in our previous study was not suitable for the identification of *O*AcGD3 by FACS as well as immunocytochemistry procedures. Besides the properties and specificity of anti-ganglioside antibodies, the accessibility of the target is also a major limitation since gangliosides are located in lipid rafts, surrounded by cholesterol and glycoproteins enhancing the steric hindrance, and the accessibility of gangliosides at the surface of BC cells may be an issue. Furthermore, antibodies targeting *O*-acetylated species may cross react and detect more than one species at a time like 493D4 [25], Jones antibody [29], A2B5 [30] targeting either *O*AcGD3/*O*AcGT3 either *O*AcGT2.

Brain tissue is the major biological material used for the analysis of *O*-acetylated ganglioside species. For example, *O*AcGT2 has been detected in cod brain by TLC and mass spectrometry combining mild alkaline treatment [31]. In bovine brain, *O*AcGD3 and *O*AcGT3 were identified as major acetylated species [25], and enhanced *O*AcGT3 was also identified in cultured glial cells [30]. *O*AcGD3 is also considered as an oncofetal marker in human melanoma [32]. The presence of *O*AcGD3 was also reported in tumors from neuroectodermal origin [33], childhood lymphoblastic leukemia [34], and glioblastoma [35]. The expression of *O*-acetylated gangliosides was poorly studied in breast cancer; a single article reports *O*AcGD3 and *O*AcGT3 expression in BC tissues using GMR2 and 493D4 mouse antibodies and TLC analysis [36]. However, some ambiguity remains because *O*-acetylated gangliosides analysis in cells and tissues was mostly performed using antibodies more or less specific of *O*-acetylated forms, some of them cross reacting with *O*-acetylated and non-*O*-acetylated ganglioside species. Here, *O*AcGM1, *O*AcGD3, *O*AcGD2, *O*AcGT2, *O*AcGT3 were identified as native gangliosides by mass spectrometry in neuroectoderm derived-cell lines. Besides, MS/MS fragmentation defined that *O*-acetylation occurs on both inner or terminal sialic acid residues. Furthermore, Neu5,9Ac_2_ was the main sialic acid species expressed among glycolipids, confirming our previous study [12]. *O*-acetylated gangliosides species were fully characterized in terms of ganglioside species and position of *O*-acetylated group on the sialic acid residues, revealing a cell-type dependent profile. Interestingly, clones over-expressing GD3 synthase showed an increased expression of complex non-acetylated and *O*-acetylated gangliosides, highlighting again the key role of GD3 synthase for b- and c-series gangliosides expression. Substrate availability is the second parameter, which drives *O*-acetylated gangliosides expression. If high amounts of precursor gangliosides are expressed, they can potentially serve for the biosynthesis of *O*-acetylated gangliosides.

*O*-acetylation of sialic acid is highly dependent on the balance between SOAT and Sialyl-*O*-acetyl-esterase (SIAE) activity. These activities are finely tuned processes, resulting in a cell type dependent pattern of *O*-acetylated gangliosides species. Recently, Mlinac et al. suggested that SIAE-induced deacetylation of GD3 increases medulloblastoma sensitivity to etoposide. The concept used in the paper was based on recovering the pro-apoptotic role of mitochondrial GD3 by deacetylation of *O*AcGD3. Besides, they showed a higher expression of SIAE in medulloblastoma compared to normal cerebellum [37]. These data seem contradictory, but they give an important insight on the complexity of the processes regulating the expression and the role of *O*AcGD3 and GD3 in cancer tissues. SOAT activity was closely related to *O*-acetylated gangliosides expression. Indeed, reduced SOAT activity decreased *O*-acetylated gangliosides, while increased SOAT activity upregulated *O*-acetylated gangliosides expression [38,39]. Despite all the attempts made for the purification of SOAT, a single human SOAT encoded by *CASD1* has been identified [40]. CASD1 is Golgi spanning multimembrane protein, which induces the *O*-acetylation of CMP-sialic acid and is involved in GD3 *O*-acetylation [13]. Here, we show that *O*-acetylation of gangliosides is not dependent on the level of CASD1, but much more dependent on the availability of ganglioside substrate and acetyl-CoA availability as underlined by the acetyl-CoA transporter SLC33A1 upregulation, regardless of the BC cell lines. However, the validation of this concept would require transfection experiments to modulate the expression of SLC33A1 or CASD1 in these cell lines.

This work highlights the cell-type dependent pattern of *O*-acetylated gangliosides species expressed by neuroectoderm-derived cell lines. Performing similar analyses in normal and cancer tissues is the next essential step to determine to what extent specific *O*-acetylated gangliosides are tumor-specific antigens and promising targets for neuroectoderm-derived tumors immunotherapy.

## 4. Materials and Methods

### 4.1. Cell Culture

The human breast cancer cells Hs 578T, MDA-MB-231, MCF-7, SUM159PT, and the melanoma cell line SK-MEL-28 were obtained from the American Tissue Culture Collection (ATCC, Rockville, MD, USA). The neuroblastoma cell line LAN-1 was obtained by Deutsche Sammlung von Mikroorganismen und Zellkulturen Gmbh (DSMZ, Leibniz Institute, Braunschweig, Germany). LAN-1 cells were cultured and maintained in Rosewall Park Memorial Institute medium 1640 (RPMI) containing 10% heat-inactivated fetal calf serum, 2 mmol/L L-glutamine, and 100 units/mL penicillin-streptomycin. MCF-7 and MDA-MB-231 clones over-expressing GD3 synthase were obtained as described [41]. All BC cell lines and the melanoma cell line SK-MEL-28 were cultured as previously described [12].

### 4.2. RNA Extraction and Quantitative Real Time Polymerase Chain Reaction

The extraction of total RNA from different cell lines was performed using Nucleospin RNA II kit (Macherey-Nagel, Düren, Germany). Extracted RNA was quantified using DeNovix DS-11 spectophotometer (DeNovix Inc., Wilmington, DE, USA). Reverse transcription of purified RNA was performed using the Maxima First Strand cDNA Synthesis Kit (Thermo Fisher Scientific, Villeneuve d’Ascq, France) according to the protocol provided by the manufacturer. The oligonucleotide sequences (Eurogentec, Seraing, Belgium) used for PCR reactions are the following primer pairs: 5′-ctg-gga-gga-aac-tgg-cct-tc-3′ and 5′-agg-gct-gta-aca-cat-gag-cc-3′ (*SCL33A1*), 5′-gtg-gat-ttt-ctg-tgg-atc-c-3′ and 5′-aag-cgc-ttc-act-gct-acc-at-3′ (*CASD1*), 5′-tat-gtg-ctg-tca-gcg-tct-gct-3′ and 5′-aca-aag-aca-tcc-tct-aat-ggg-aga-a-3′ (*B3GALT4)*, 5′-gtg-gat-ttt-ctg-tgg-cat-cc-3′ and 5′-aag-gcg-ttc-act-gct-acc-at-3′(*ST8SIA5*). PCR reactions were processed using Mx3005p Quantitative System (Stratagene, La Jolla, CA, USA) using 2X Brilliant SYBR Green qPCR Mastermix (Thermo Fisher Scientific, Villeneuve d’Ascq, France) in 300 nM of primers and 4 µL cDNA as previously described [12]. All experiments were performed in triplicate.

### 4.3. DMB Derivatization of Sialic Acids

Cells were suspended in PBS and centrifuged 15 min at 4000 rpm. Pellets were sequentially extracted by CHCl_3_/CH_3_OH (2/1; *v*/*v*) and CHCl_3_/CH_3_OH/H_2_O (1/2/0.8; *v*/*v*/*v*). Pellets contained membrane associated glycoproteins, whereas supernatants contained the ganglioside fractions. Supernatants were precipitated with ice cold 100% ethanol overnight and centrifuged 5 min at 10,000× *g*. Supernatants contained free sialic acids, whereas pellets contained soluble proteins [42]. Dried pellets of BC cell lines were hydrolyzed at 80 °C for 4 h in 4 M propionic acid and then precipitated in 4 volumes of 100% ethanol. Hydrolyzed sialic acids were subsequently coupled to 1,2-diamino-4,5-methylenedioxybenzene dihydrochloride (DMB). Samples were heated at 50 °C for 2 h in the dark in 7 mM DMB, 1 M β-mercaptoethanol, 18 mM sodium hydrosulfite in 5 mM acetic acid [43]. Sialic acids coupled to DMB (DMB-Sia) were then analyzed by LC-MS.

### 4.4. Quantitation Analysis of DMB-Sia on Micro-LC/ESI-MS3

Quantitative analyses were performed in positive ion mode on an amaZon speed ETD ion trap mass spectrometer equipped with the standard electrospray ionization (ESI) ion source and controlled by Hystar 3.2 software (Bruker Daltonics, Billerica, MA, USA). DMB-coupled sialic acid separation was achieved on micro LC system (Prominence LC-20AB, Shimadzu, Kyoto, Japan). 5 µL of samples were applied to the reversed-phase Luna C18-2 column (150 × 1.00 mm, 3 µm particles, Phenomenex, Torrance, CA, USA) with an isocratic elution of CH_3_CN/CH_3_OH/H_2_O (6/4/90; *v*/*v*/*v*) at a flow rate of 70 µL/min. The targeted MS3 scans for DMB-coupled sialic acid were performed using an ultrascan mode (26,000 amu/s). Data obtained for external standards run on the same time were used for estimation of DMB-Sia amounts in BC cell line our BC cell lines samples. Sialic acid species were identified by referring to elution positions and MS_3_ fragmentation of Neu5Ac and Neu5,9Ac_2_ standards. The reported values were based on signal area of the single ion chromatogram at the appropriate retention time [44].

### 4.5. Extraction of Native Gangliosides

Cell were suspended in PBS and centrifuged at 4000 rpm during 15 min. Pellets were extracted thrice by 2 volumes of CHCl_3_/CH_3_OH (2/1; *v*/*v*) and 1 volume CHCl_3_/CH_3_OH/H_2_O (1/2/0.8; *v*/*v*/*v*). Supernatants were collected and dried gently under N_2_ stream. Glycosphingolipids were separated from other lipids and from hydrophilic components on a tC_18_ cartridge Sep-Pak connected to QMA Sep-Pack cartridge (Waters, St Quentin Yvelines, France) equilibrated in CH_3_OH/CF_3_COOH/H_2_O (1/0.1/1; *v*/*v*/*v*) by extensive washing. Neutral glycosphingolipids were eluted in 2 volumes of CH_3_OH. Acidic glycosphingolipids were eluted twice in CH_3_OH/CH_3_COONH_4_ (1/1; *v*/*v*) within 0.05 M, 0.15 M, and 0.45 M CH_3_COONH_4_ sequentially in 1 volume each. Neutral and acidic glycosphingolipids were dried gently under N_2_ stream. Acidic glycosphingolipids were separated from ammonium acetate on a tC_18_ cartridges Sep-pack (Waters, St Quentin Yvelines, France) equilibrated in CH_3_OH/CF_3_COOH/H_2_O mixture (1/0.1/1; *v*/*v*/*v*) by extensive washing. Acidic glycosphingolipids were eluted in 2 volumes of CH_3_OH and dried under N_2_ stream before reconstitution in CHCl_3_/CH_3_OH (1/2; *v*/*v*) and analysis on MALDI-QIT-TOF.

### 4.6. Mass Spectrometry Analysis

Acidic glycosphingolipids were analyzed by an MALDI-QIT-TOF Shimadzu AXIMA Resonance mass spectrometer (Shimadzu Europe, Manchester, UK) in the negative mode. Samples were prepared by mixing directly on the target 0.5 µL of acidic glycosphingolipid sample with 0.5 µL 2′-4′-6′-trihydroxyacetophenone monohydrate matrix solution (0.5 M in EtOH) containing by 0.1 M hydrated di-ammonium hydrogen citrate mixture (2/1; *v*/*v*). The mid mode for a mass range *m*/*z* 1500–3000 was used and laser power was set to 100 2 shots each in 200 locations per spot.

### 4.7. In Silico Mapping of Glycosyltransferase Gene Expression and Ganglioside Species Quantification

The pathway was retrieved from WikiPathways [18] using the WikiPathways app [19] for Cytoscape [45], the “Ganglio Sphingolipid Metabolism” pathway for *Homo sapiens* was used as a basis. Only a subpart was conserved and enriched with some metabolites and genes. For each figure, qPCR data were mapped onto the genes of the pathway, calculating the log2 ratio of expression of one condition and a second one; colors vary from blue (≤ −2) to white (= 0) and red (≥ 2). Relative quantity amounts of gangliosides measured in MALDI-QIT-TOF mass spectroscopy were also mapped onto the gangliosides in the pathway, calculating the difference in signals between two conditions; colors vary from green (≤ −8) to white (= 0) and fuchsia (≥ 8).

## Figures and Tables

**Table 1:** List of identified gangliosides in neuroectoderm derived cells. Monosylalylated GSL are observed at [M−H]^−^. Di- and trisialylated GSL were mainly observed at *m*/*z* [M−H_2_O−H]^−^ and [M−2H_2_O−H]^−^. √ indicates the presence of GSL, and √ T indicates that GSL is present in traces amount.

GSL	Fatty Acids	Monoisotopic Mass [M-H]-*m*/*z*	Lactonization	SK-Mel-28	LAN-1	SUM 159 PT	Hs 578T	MDA-MB-231	MDA-MB-231 GD3S+ Clone #4	MDA-MB-231 GD3S+ Clone #11	MCF-7	MCF-7 GD3S+
GM3	C16	1151.7		√	√			√	√	√		
	C18	1179.7		√								
	C22	1235.8		√				√		√		
	C24	1263.8		√			√	√	√	√		
GM2	C16	1354.8		√	√	√	√	√	√	√	√	√
	C18	1382.8			√							
	C22	1438.9			√	√	√	√	√	√	√	√
	C24	1466.9			√	√	√	√	√	√		
GM1	C16	1516.8			√	√	√	√	√	√	√	
	C24	1629.0		√	√	√	√	√		√	√	√
LacNAcGM1	C16	1882.0			√							
GD3	C16	1424.8	-H_2_O	√	√							
	C16	1442.8		√								
	C18	1452.8	-H_2_O	√								
	C22	1508.9	-H_2_O	√						√		
	C24	1536.9	-H_2_O	√					√	√		
	C24	1554.9		√					√	√		
GD2	C16	1628.9	-H_2_O		√	√	√	√T	√	√		√T
	C18	1671.9		√	√				√	√		
	C24	1740.0	-H_2_O			√	√		√	√		
GD1b	C24	1902.1	-H_2_O						√	√		
GT3	C24	1810.0	-2H_2_O						√	√		
GT2	C24	2013.1	-2H_2_O						√	√		
*O*AcGM1	C24	1671					√		√			
*O*AcGD3	C14	1438.8	-H_2_O			√	√T		√	√		
	C16	1466.8	-H_2_O	√		√	√T					
	C24	1578.9	-H_2_O	√						√		
*O*AcGD2	C16	1670	-2H_2_O		√	√	√			√		√
	C24	1782	-H_2_O						√	√		
*O*AcGT3	C16	1740.0	-2H_2_O						√T	√		√
	C24	1852.0	-2H_2_O						√	√		
*O*AcGT2	C24	2055.1							√	√		

## Supplementary Materials

Supplementary Materials can be found at https://www.mdpi.com/1422-0067/21/1/370/s1.

## Abbreviations

BC	Breast Cancer
GlcCer	Glucosylceramide
LacCer	Lactosylceramide
LC-ESI/MS	Liquid Chromatography Electrospray Ionisation/Mass Spectrometry
Neu5,9Ac_2_	9-*O-*acetyl-N-acetylneuraminic acid
*O*AcGD2	*O*-acetylated GD2
*O*AcGD3	*O*-acetylated GD3
RPLC-MS	Reverse-Phase Liquid Chromatography Mass Spectrometry
SCLC	Small Cell Lung Carcinoma
SOAT	Sialyl-*O*-acetyltransferase
SIAE	Sialyl-*O-*acetylesterase
TACA	Tumor Associated Carbohydrate Antigen
TLC	Thin Layer Chromatography
