# Supplementary material for: Profiling of O-acetylated Gangliosides Expressed in Neuroectoderm Derived Cells
Source: Int J Mol Sci. 2020 Jan 6;21(1):370. doi: 10.3390/ijms21010370 (PMC6981417; doi:10.3390/ijms21010370)

Supplementary Figure S1: Representative ganglioside profile of SK-Mel-28 (A), LAN-1 (B), SUM 159 PT (C). Ceramide consists mainly of d18:1 long chain base and C16, C18, C24 either/both saturated or/and saturated fatty acids. The mass number with ceramide length colored in blue means the structure were identified by MS_2 fragmentation. The mass number colored in red indicates the presence of *O*-Acetylated structures. * indicates that signal corresponds to a mixture of GM1Cer[24] and GD2Cer[16] (C). Gangliosides are indicated according to the following nomenclature Ceramide; Galactose; Glucose; N-acetyl-galactosamine; N-acetyl-neuraminic acid, Ac: O-acetyl group.

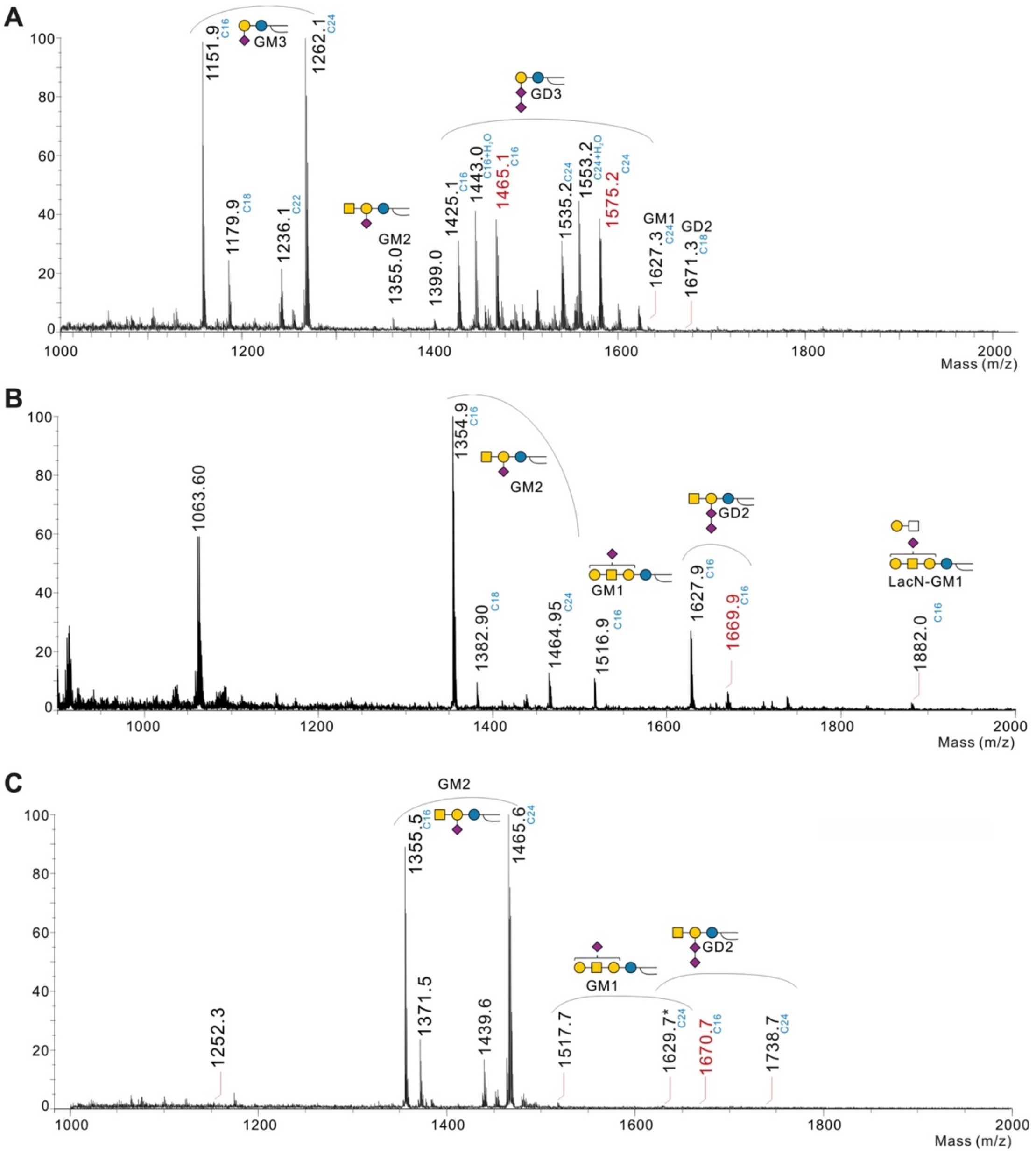

Supplementary Figure S1 continued: Representative ganglioside profile of MDA-MB-231 (D), MDA-MB-231 GD3S+ clone #11 (E), MCF-7 (F), MCF-7 GD3S+ (G). Ceramide consists mainly of d18:1 long chain base and C16, C18, C24 either/both saturated or/and saturated fatty acids. The mass number with ceramide length colored in blue means the structure were identified by MS_2 fragmentation. The mass number colored in red indicates the presence of *O*-Acetylated structures. * indicates that signal corresponds to a mixture of GM2Cer^{16} with *O*AcGM3Cer^{14}; ** indicates that signal corresponds to a mixture of GM1Cer^{24} and GD2Cer^{16}; *** indicates that signal corresponds to a mixture of *O*AcGD2Cer^{16} and GD2Cer^{18}; **** indicates that *O*AcGT3Cer^{16} is present in traces amount (E). a, b, c, d, e, f indicates that ceramide is highly hydroxylated (F-G). Gangliosides are indicated according to the following nomenclature: Ceramide; Galactose; Glucose; N-acetyl-galactosamine; N-acetyl-neuraminic acid, Ac: O-acetyl group.

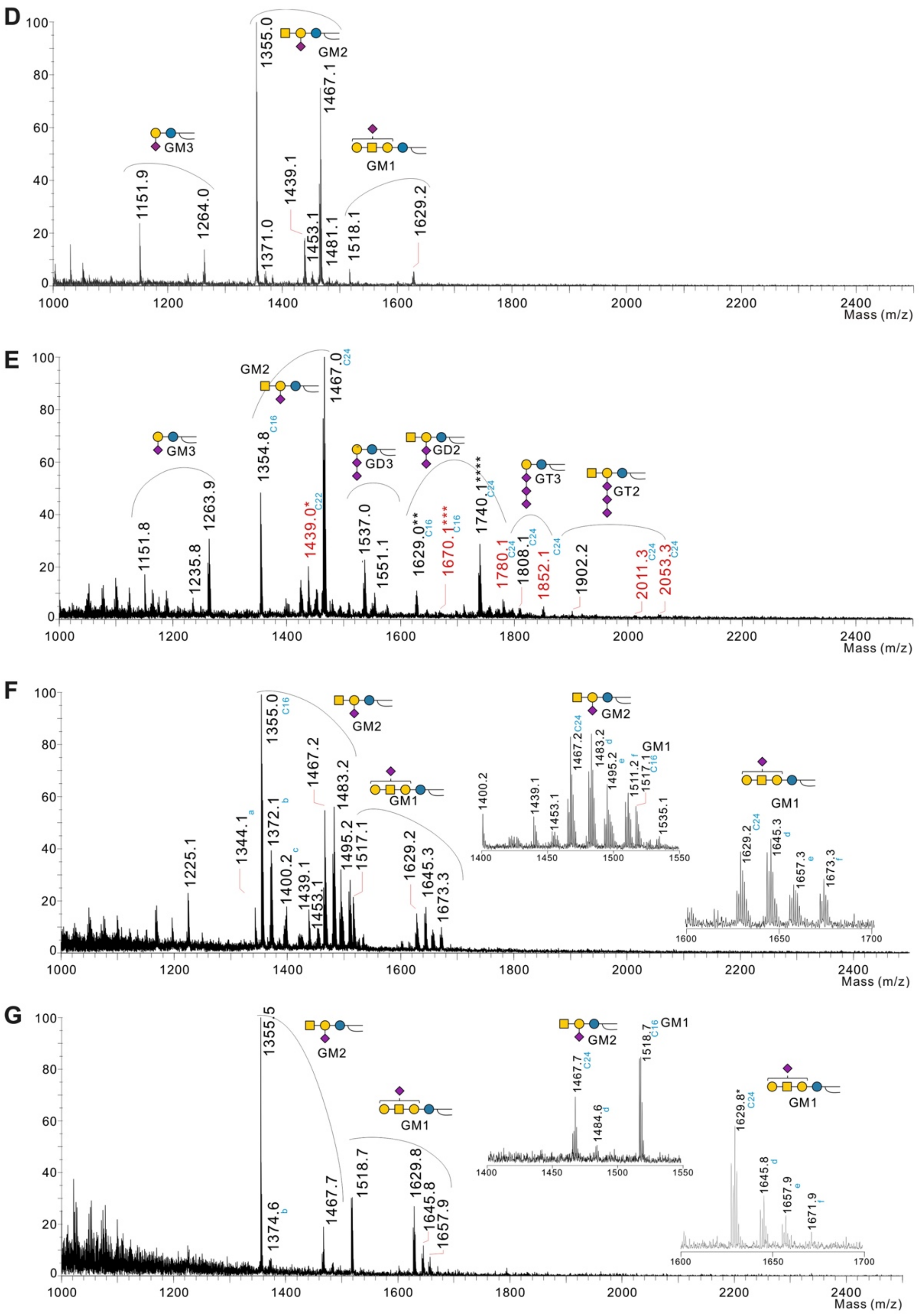

Supplementary Figure S2: Glycosyltransferase gene expression profiling in neuroectoderm derived cancer cell lines. *B4GALT6* (Lactosylceramide synthase), *ST3GAL5* (GM3 synthase), *ST8SIA1* (GD3 synthase), *B4GALNT1* (GD2 synthase), *ST8SIA5* (GT3 synthase) by qPCR. Gene expression analysis of *CASD1* encoding for SOAT, and *SCL33A1* encoding for Acetyl CoA transporter by qPCR on the same cell lines. Each bar represents the mean ± SD of n= 3 experiments.

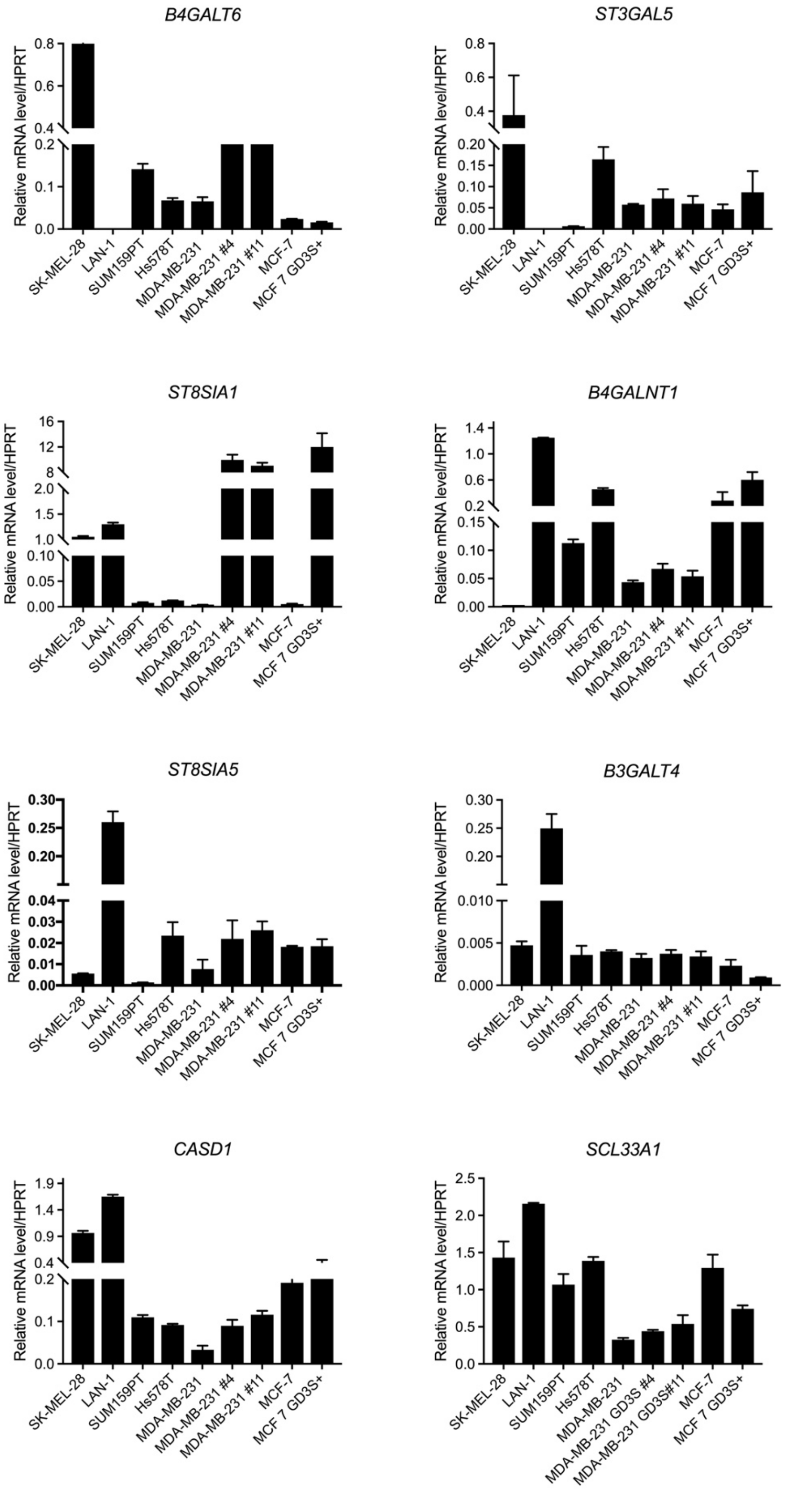

Supplementary Figure S3: Differential ganglioside metabolism pathway between Hs 578T *vs* MCF-7 (A) and MDA-MB-231 GD3S+ clone #11 *vs* MDA-MB-231 (B). Glycosyltransferase gene expression data obtained by qPCR were mapped onto Wikipathway [27,28] based on the differential expression between two cell lines. In the squared nodes, colors vary from blue to red for indicating the repression to the over-expression of the glycosyltransferase gene in the first cell line compared to the second which are pointed by the thick black arrow. Quantitative data concerning the amounts of gangliosides obtained by MALDI-QIT-TOF mass spectrometry were added to the pathway based on the comparison between the first and the second cell line. In the octagonal nodes, colors vary from green to fuchsia to indicate a restraint to a rise of the amount of a given ganglioside based on the difference between the two cell lines. Grey color indicates the absence of any available data about the expression

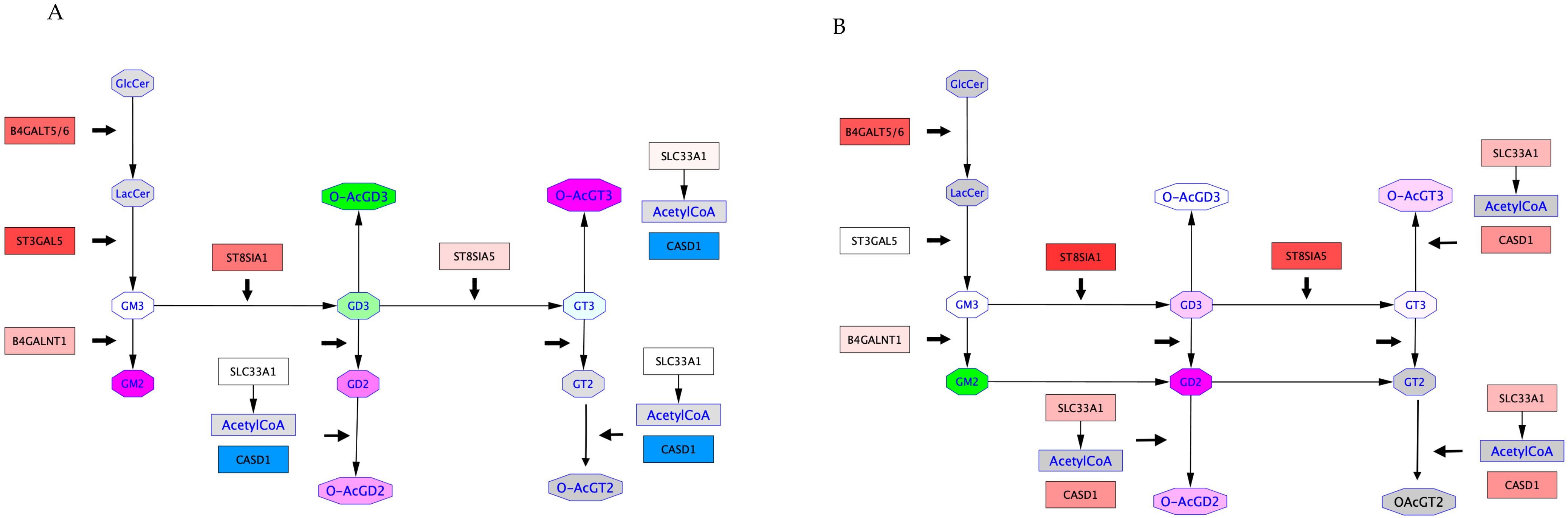

Supplement: Supplementary file 1 [file ijms-21-00370-s001.pdf]
